# Viscoelastic Relaxation of Polymerized Ionic Liquid and Lithium Salt Mixtures: Effect of Salt Concentration

**DOI:** 10.3390/polym13111772

**Published:** 2021-05-28

**Authors:** Arisa Yokokoji, Wakana Kitayama, Kamonthira Wichai, Osamu Urakawa, Atsushi Matsumoto, Visit Vao-Soongnern, Tadashi Inoue

**Affiliations:** 1 Department of Macromolecular Science, Graduate School of Science, Osaka University, 1-1 Machikaneyama-cho, Toyonaka, Osaka 560-0043, Japan; yokokoujia19@chem.sci.osaka-u.ac.jp (A.Y.); kitayamaw18@chem.sci.osaka-u.ac.jp (W.K.); kamonthiraw20@chem.sci.osaka-u.ac.jp (K.W.); 2 School of Chemistry, Institute of Science, Suranaree University of Technology, Nakon Ratchasima 30000, Thailand; visit@sut.ac.th; 3 Department of Applied Chemistry and Biotechnology, University of Fukui, 3-9-1 Bunkyo, Fukui-shi, Fukui 910-8507, Japan; atsushi5@u-fukui.ac.jp

**Keywords:** rheology, polymerized ionic liquid, lithium salt, glass transition temperature, sub-Rouse mode, plateau modulus, WLF, iso-frictional state

## Abstract

Polymerized ionic liquids (PILs) doped with lithium salts have recently attracted research interests as the polymer component in lithium-ion batteries because of their high ionic mobilities and lithium-ion transference numbers. To date, although the ion transport mechanism in lithium-doped PILs has been considerably studied, the role of lithium salts on the dynamics of PIL chains remains poorly understood. Herein, we examine the thermal and rheological behaviors of the mixture of poly(1-butyl-3-vinylimidazolium bis(trifluoromethanesulfonyl)imide (PC_4_-TFSI)/lithium TFSI (LiTFSI) in order to clarify the effect of the addition of LiTFSI. We show that the glass transition temperature *T*_g_ and the entanglement density decrease with the increase in LiTFSI concentration $w_{\mathrm{LiTFSI}}$. These results indicate that LiTFSI acts as a plasticizer for PC_4_-TFSI. Comparison of the frequency dependence of the complex modulus under the iso-frictional condition reveals that the addition of LiTFSI does not modify the stress relaxation mechanism of PC_4_-TFSI, including its characteristic time scale. This suggests that the doped LiTFSI, component that can be carrier ions, is not so firmly bound to the polymer chain as it modifies the chain dynamics. In addition, a broadening of the loss modulus spectrum in the glass region occurs at high $w_{\mathrm{LiTFSI}}$. This change in the spectrum can be caused by the responses of free TFSI and/or coordination complexes of Li and TFSI. Our detailed rheological analysis can extract the information of the dynamical features for PIL/salt mixtures and may provide helpful knowledge for the control of mechanical properties and ion mobilities in PILs.

## 1. Introduction

Polymerized ionic liquids (PILs), a subclass of solid polymer electrolytes, have advantageous properties of ionic liquids, such as flame-retardant, wide electrochemical window, and chemical stabilities, and thus can serve as a promising material for next-generation solid-state batteries [1,2,3,4,5,6]. PILs bear a charged group with ionic liquid structures in their main chain and/or side chain, weakly coordinated with oppositely charged counterions. One of the significant challenges for the practical use of polymers, such as PILs, as a solid electrolyte is to generate high ionic conductivities of liquid-based electrolytes while maintaining their robust mechanical properties [2,7,8,9,10]. Accordingly, understanding the dynamics of polymer chains and ions and their correlations is vital in designing PIL-based materials with high performance.

As the first attempt for solid polymer electrolytes, poly(ethylene oxide) (PEO)/lithium (Li) salt mixtures are intensively examined [3,11,12,13,14,15,16]. These studies have revealed the following ion transport mechanism: the transport of Li ions occurs cooperatively with the segmental motion of polymer chains due to the ether oxygen–Li^+^ coordination. As a result, both ionic conductivity and Li^+^ transference number in PEO/Li salt mixtures become typically low due to the limitation of Li^+^ movements by the interaction with PEO chains [3,17]. Thus, the acceleration of the segmental motion, i.e., lowering glass transition temperature *T*_g_, is necessary to increase their ionic conductivities.

When adding Li salts into PILs, recent experimental and theoretical studies [18,19] have shown that both ionic mobility and Li^+^ transference number increase with increasing salt concentrations, opposite to the trend of PEO/Li salt mixtures. The improvement in ionic conductivity was attributed to the decrease in the glass transition temperature of PIL/Li salt mixtures with increasing salt concentrations, caused by the modification of coordinated structures between polyions, counterions, and Li-ions [18,19,20]. These results indicate that the mixture of PILs and Li salts can be an appealing solid-state polymer electrolyte alternative to conventional polyether/Li-salt mixtures. To date, although the ion transport in lithium-doped PILs has been considerably investigated, the role of lithium salts on the dynamics of PIL chains remains poorly understood. Hierarchical modes exist in polymer chain dynamics. The slow chain mode determines the flowability, the entanglement strand motion is related to the rubber elasticity, and the short-time segmental dynamics governs the transport of ions in mixtures with ionic species. Therefore, understanding the polymer dynamics is of great importance to optimize the PIL’s mechanical and electrical properties.

**Figure 1:**
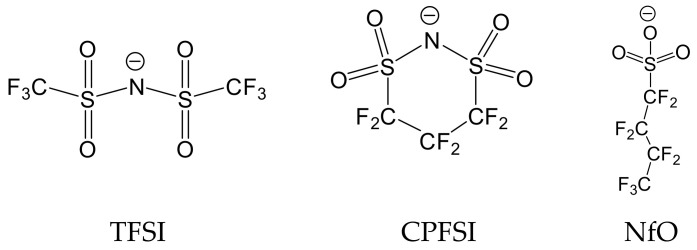
Chemical structures of TFSI, CPFSI, and NfO anions.

Nakamura and co-workers examined the viscoelastic spectrum of several kinds of PILs spanning from the glass to terminal regions [21,22,23,24,25]. They observed a specific relaxation in the glass-to-rubber transition region when the counterions are relatively large in sizes, such as bis(trifluoromethane sulfonyl imide) (TFSI), 1,1,2,2,3,3-hexafluoropropane-1,3-disulfonimide (CPFSI), and nonafluorobutane sulfonate (NfO), whose structures are shown in Figure 1 [24,25]. Based on the analysis using the modified stress-optical rule, they proposed that the observed specific relaxation is attributable to the sub-Rouse mode, which has long been known to appear in bulk polyisobutylene (PIB) [26,27,28] and polymer solutions [29]. Weak intermolecular coupling [30] and strong intramolecular cooperativity of chain segments [28,29,31] have been considered to be the sub-Rouse mechanism. Inoue et al. demonstrated that the damped torsional oscillator model could successfully describe the sub-Rouse mode of the PIL system and concluded that the origin of this relaxation is the cooperative torsional motions of structural units in a polymer chain [25].

**Figure 2:**
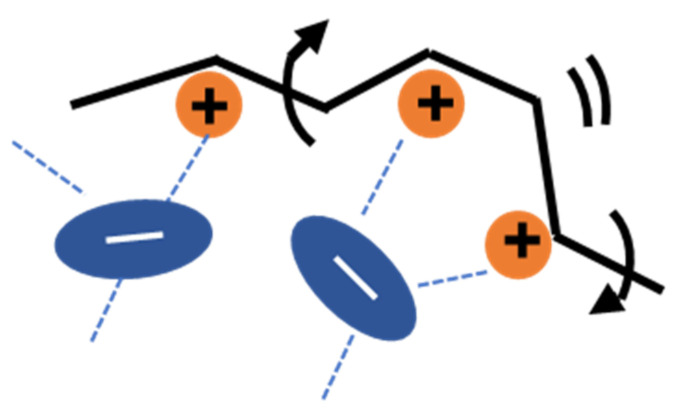
Schematic representation of the molecular motion responsible for the sub-Rouse mode. The free segmental movement of polymer chains is restricted by the interaction between the counter anion and polymer chain cations.

For PILs having large-size counterions, as shown in Figure 1, the Coulomb interaction is relatively weak due to the charge delocalization in these anions since CF_3_ and CF_2_ are strong electron-withdrawing groups. As a result, the degree of dissociation becomes high, and the dissociated anion can behave as solvent (diluent) molecules to reduce the interchain coupling. Such dilution effects can be further supported by the fact that the glass transition temperature is much lower for PILs with TFSI and CPFSI than those with smaller counter-anions, such as BF_4_ [22]. The dilution of PIL chains by the dissociated anion can lead to the appearance of the intramolecular mode (sub-Rouse mode). NfO has a molecular size comparable to TFSI but has a structure with negative charges concentrated on only one side. From this feature, the anionic side of a NfO molecule bind to the polymer cation with higher strength than TFSI and CPFSI, and thus the solvent-like behavior (dilution effect) may not be readily apparent. Nevertheless, sub-Rouse relaxation was observed in the NfO system [24]. The stress-optical coefficient obtained by the rheo-optical measurements for this system indicated that the sub-Rouse mode is caused by the local motion of the polymer chain accompanied by the motion of the NfO anion. This result, therefore, suggests that the restriction of the segmental movement by the strongly bonded NfO makes the polymer chain stiffer and enhances the torsional sub-Rouse motion. We also assume that the interchain bonding via the NfO anion is unlikely to occur because the negatively charged coordination site, SO^3−^, is screened by the outer fluorocarbon segment, reducing the interchain cooperativity. In summary, in PILs with NfO anions, the effects of anion coordination and the shielding of intermolecular interactions make the sub-Rouse mode more pronounced. Recently, Zhang et al. performed MD simulations on a PIL/Li salt system and indicated that even the charge-delocalized TFSI anions could coordinate with polymer cations as schematically shown in Figure 2 [19]. From their simulation result, we assume that the intramolecular coordination structures depicted in Figure 2 can increase intramolecular cooperativity and possibly emphasize the sub-Rouse mode. Based on the above argument, we anticipate that polymer chain dynamics will be affected by changing the association structure of the counter ions to the polymer chain.

In this study, we investigate how the addition of Li salts (LiTFSI) with various concentrations affects the thermal and dynamical features in a typical PIL, poly(1-butyl-3-vinyl imidazolium bis(trifluoromethane sulfonyl)imide) (PC_4_-TFSI) by means of small amplitude oscillatory shear rheometry and differential scanning calorimetry measurements. How the hierarchical dynamics of PIL is affected by changes in the Coulomb interaction is of significant academic interest. We believe that a deeper understanding of the PIL dynamics will provide insights for improving the ionic conductivity and mechanical properties of PIL/Li-salt materials.

## 2. Materials and Methods

### 2.1. Materials

1-vinylimidazole and 1-bromobutane were purchased from Tokyo chemical industry Co., Ltd. (Tokyo, Japan). Lithium bis(trifluoromethanesulfonyl)imide (LiTFSI), 2,2′-azobis(isobutyronitrile), silver nitrate (AgNO_3_), acetone, and methanol were obtained from FUJIFILM Wako Pure Chemical Corporation (Osaka, Japan). Deuterium oxide (D_2_O) was purchased from Cambridge Isotope Laboratories, Inc. (Massachusetts, USA) as a solvent for NMR measurement. Methanol was used after distillation. Other chemicals were used as received. DI water was obtained by using Elix system (Tokyo, Japan).

### 2.2. Synthesis and Characterization of Materials

**Figure 3:**
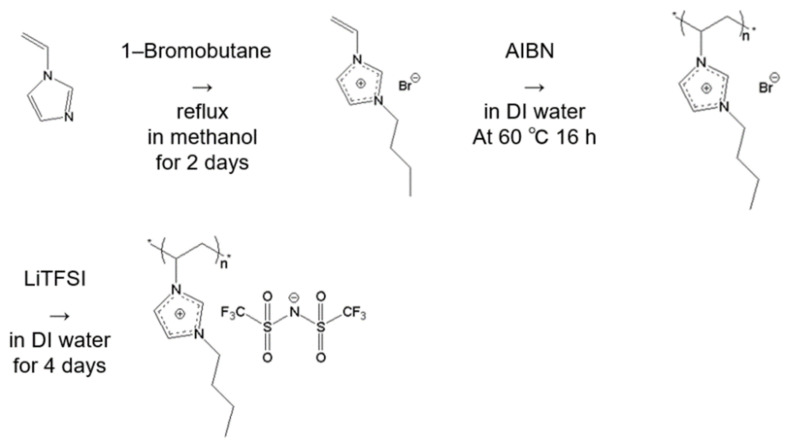
The step for synthesis of poly(1-butyl-3-vinylimidazolium bis(trifluoromethanesulfonyl) imide (PC_4_-TFSI).

The synthesis of poly(1-butyl-3-vinylimidazolium bis(trifluoromethanesulfonyl)imide (PC_4_-TFSI) was carried out in the same method as reported previously [21,22,23,25] (see Figure 3). 1-Vinylimidazolium bromide (C_4_-Br) was first synthesized by quaternizing 1-vinylimidazole with the excess amount of 1-bromobutane in methanol for 2 days at 50 °C. After removing methanol and unreacted 1-bromobutane, the C_4_-Br was polymerized through free radical polymerization in DI water by using initiator AIBN for 16 h at 60 °C. Finally, PC_4_-TFSI was synthesized by converting counter anions from Br^−^ to TFSI by titrating LiTFSI aqueous solution into PC_4_-Br aqueous solution and mixed for 4 days at room temperature.

The purity of C_4_-Br and PC_4_-Br was confirmed by 1H-NMR in D_2_O and elemental analysis (Yanako CHN coder MT-6, Yanako Technical Science, Tokyo, Japan), respectively. The experimentally obtained weight ratios for C, H, and N elements were 30.88 wt% (C), 3.63 wt% (H), and 10.10 wt% (N). (The calculated ones for C_11_H_15_N_3_O_4_S_2_F_6_ are 30.62 wt% (C), 3.51 wt% (H), and 9.74 wt% (N)).

In order to estimate the molecular weight for the PC_4_-TFSI sample, first, we determined the intrinsic viscosity [*η*] for the DMF solution by the method proposed previously [32]. The zero shear viscosities (η) at two different concentrations (c = 1.59 × 10^−3^ g cm^−3^ and c = 4.76 × 10^−4^ g cm^−3^) measured with ARES G2 (TA Instruments Inc., Tokyo, Japan) were cast into the following equation [32] and estimated the [*η*] values.

(1) $$\left[\eta \right]=\frac{{\left[2\left(\eta_{sp}-ln\eta_{r}\right)\right]}^{\frac{1}{2}}}{c},\eta_{sp}=\frac{\eta -\eta_{0}}{\eta_{0}},\eta_{r}=\frac{\eta }{\eta_{0}}$$

Here, $\eta_{sp}$, $\eta_{r}$, and $\eta_{0}$ are respectively the specific viscosity, relative viscosity, and solvent viscosity (0.92 mPa s). We obtained the same $\left[\eta \right]$ values at two different *c* within the experimental error: $\left[\eta \right]=653{\;\mathrm{cm}}^{3}{\;g}^{-1}$. Using the values of molecular weight (*M*_w_ = 1.03 × 10^6^), intrinsic viscosity ($\left[\eta \right]=2958{\;\mathrm{cm}}^{3}{\;g}^{-1}$ equally estimated from the zero shear viscosity), and the Flory exponent (*ν* = 0.75) reported by Matsumoto et al. [33] as reference data, we determined the molecular weight of the PC_4_-TFSI to be *M*_w_ = 3.0 × 10^5^ using the relation of $1.03\times 10^{6}{\left(\frac{653}{2958}\right)}^{1/\left(3\nu -1\right)}$.

### 2.3. Blend Sample Preparation

The mixtures of PC_4_-TFSI/LiTFSI were prepared by mixing the prescribed amount of the components in acetone, and then the solvent acetone was removed by the solvent casting method at room temperature. The weight fractions of LiTFSI ($w_{\mathrm{LiTFSI}}$) in the PC_4_-TFSI/LiTFSI mixtures were 0.039, 0.070, 0.11, 0.15, 0.20, 0.25, 0.30, 0.35, 0.40, and 0.50. After solvent casting, the sample films were dried at 100 °C under vacuum and then molded into a flat sheet by the hot press at temperatures in the range of 120–150 °C depending on the glass transition temperature for each salt concentration. Since LiTFSI is hygroscopic, the sheets were dried at 100 °C under vacuum again to remove the absorbed water before use. We confirmed that the blend samples with $w_{\mathrm{LiTFSI}}$ ≤ 0.3 were transparent, but the samples with $w_{\mathrm{LiTFSI}}$ ≥ 0.4 were slightly opaque.

### 2.4. Measurements

The complex shear modulus G* = *G*′ + i*G*″, where *G*’ is the storage modulus and, *G*″ is the loss modulus, in a frequency range *ω* from 0.1 to 100 s^−1^ was measured using ARES G2 (TA Instruments Inc.) equipped with home-made 4 mm parallel plate fixtures under oscillatory shear deformation (*γ* = 0.01–30%) by following the ISO 6721-10 and JIS K7244-10. A sample was placed on the lower plate, and the distance between the upper and lower plates was reduced at about 100 °C so that the sample was sandwiched and completely filled between the two plates. The sample shape was 4 mm in diameter and 0.7~2.0 mm in thickness. The strain amplitudes were set to be in the linear regime for each temperature determined using the amplitude sweep test at ω = 1 s^−1^. We examined the *ω* dependence of G* in the temperature range from 10 to 180 °C under a nitrogen atmosphere.

The calorimetric glass transition temperature *T*_g_ for all the samples was determined with a differential scanning calorimeter (DSC 6220, Seiko Instruments Inc., Chiba, Japan). The rate of both heating and cooling processes was 10 °C min^−1^. Three thermal scan cycles of 1st heating, 1st cooling, 2nd heating, 2nd cooling, and 3rd heating were conducted in the temperature range of −20 °C–180 °C. We determined *T*_g_ from the 2nd heating scan as the temperature at which the time derivative of the heat flow curve exhibited a peak. We also confirmed that the 3rd heating data matched the second data for each sample.

To examine the miscibility of the PC_4_-TFSI/LiTFSI mixtures, we conducted optical microscopy observation for all the samples sandwiched between the glass slide and the cover glass with a spacer of 0.2 mm thickness using a polarizing and bright field microscope, E400 (Nikon, Tokyo, Japan).

## 3. Results and Discussion

### 3.1. Miscibility of PC_4_-TFSI/LiTFSI Mixtures

**Figure 4:**
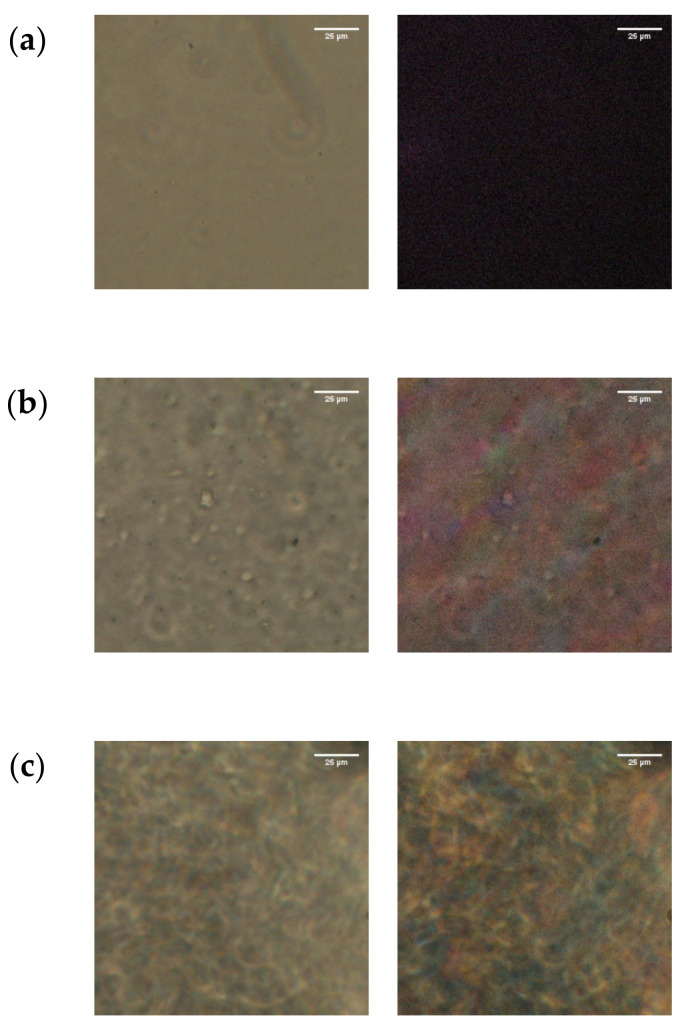
Optical micrographs for the PC_4_-TFSI/LiTFSI mixtures with $w_{\mathrm{LiTFSI}}$ = 0.3 (**a**), 0.4 (**b**), 0.5 (**c**). The left- and right-hand pictures are respectively the bright-field micrographs and the cross-polarized micrographs, in which the polarizer and the analyzer were set in horizontal and vertical directions, respectively. The scale bar in each figure is 25 μm.

Figure 4 shows the typical bright-field and cross-polarized micrographs (left and right, respectively) of the PC_4_-TFSI/LiTFSI mixtures with $w_{\mathrm{LiTFSI}}$ = 0.3, 0.4, and 0.5. At $w_{\mathrm{LiTFSI}}$ = 0.3, no phase-separated structure was observed in the bright-field micrograph, and the cross-polarized micrograph is completely dark, indicating that LiTFSI is homogeneously mixed with PC_4_-TFSI. On the other hand, for the $w_{\mathrm{LiTFSI}}$ = 0.4 and 0.5 mixtures, inhomogeneous textures are observed in both the bright-field and the cross-polarized micrographs, indicating the existence of an optically anisotropic region. These results suggest that LiTFSI does not entirely dissolve in the PC_4_-TFSI, and the crystalline phase of LiTFSI exists for $w_{\mathrm{LiTFSI}}$ ≥ 0.4. The coexistence of polymer-salt amorphous and LiTFSI crystalline phases was also observed for different PIL/Li-salt systems at high salt concentrations [18]. Based on this result, we discuss the rheological behavior for only the homogeneous samples ($w_{\mathrm{LiTFSI}}$ ≤ 0.3) in this study in order to avoid the effect of crystalline structures on the dynamics of PIL chains.

### 3.2. Weight Fraction Dependence of Calorimetric Glass Transition Temperatures

**Figure 5:**
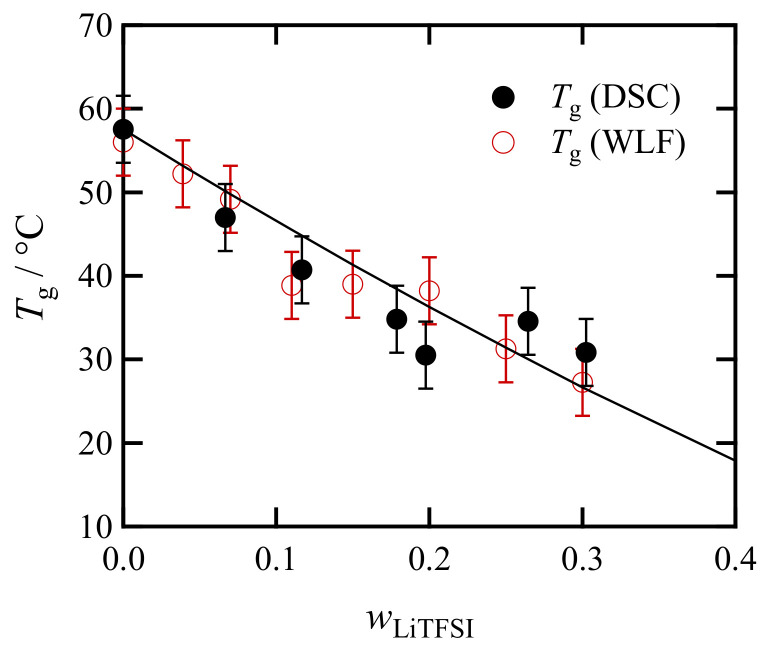
Dependence on the LiTFSI concentration for the glass transition temperatures measured by DSC (*T*_g_(DSC)) and estimated from the viscoelastic shift factors (*T*_g_(WLF)) explained in the Appendix A section. The solid curve represents the fit functions given by Equation (2).

Figure 5 shows the LiTFSI concentration ($w_{\mathrm{LiTFSI}}$) dependence of the glass transition temperature *T*_g_(DSC) for the mixture of PC_4_-TFSI and LiTFSI, obtained from the DSC measurements. The solid curve represents the fit result of the measured *T*_g_ by the following conventional Fox equation:

(2) $$\frac{1}{T_{g}}=\frac{1-w_{\mathrm{LiTFSI}}}{T_{g,{\mathrm{PC}}_{4}\mathrm{TFSI}}}+\frac{w_{\mathrm{LiTFSI}}}{T_{g,\mathrm{LiTFSI}}}$$

where $T_{g,{\mathrm{PC}}_{4}\mathrm{TFSI}}$ and $T_{g,\mathrm{LiTFSI}}$ are respectively the glass transition temperatures of pure PIL ($w_{\mathrm{LiTFSI}}=0$) and LiTFSI ($w_{\mathrm{LiTFSI}}=1$). In this curve fitting, only $T_{g,\;\mathrm{LiTFSI}}$ is a fitting parameter, and the best fit was obtained with $T_{g,\mathrm{LiTFSI}}=-26{}^{\circ}C$. Note that it is technically difficult to acquire amorphous structures of LiTFSI, and thus, we cannot estimate the value of *T*_g,LiTFSI_ using our DSC experiments. Nevertheless, since ionic liquids consisting of large cations and TFSI anions typically show glass transition temperatures around −80 °C [34], the estimated value of $T_{g,\mathrm{LiTFSI}}=-26\;{}^{\circ}C$ for LiTFSI with small cations and thus strong Coulomb interactions might be reasonable.

Regarding the monotonical decrease of *T*_g_, this trend is in good agreement with the literature data [18] and a superior point to achieve high mobility and high conductivity for Li^+^ ions. As for the mechanism, we believe that the complex change of coordinated ionic structures between polyions, counterions, and Li-ions [18,19,20] could increase the free volume. As presented in the Appendix A section, the temperature and composition dependence of the free volume fraction can be described by the free volume theory, and we expect that further details will become clearer in future discussions combined with MD simulations.

### 3.3. Overview of the Chain Dynamics in PC_4_-TFST Molten System

**Figure 6:**
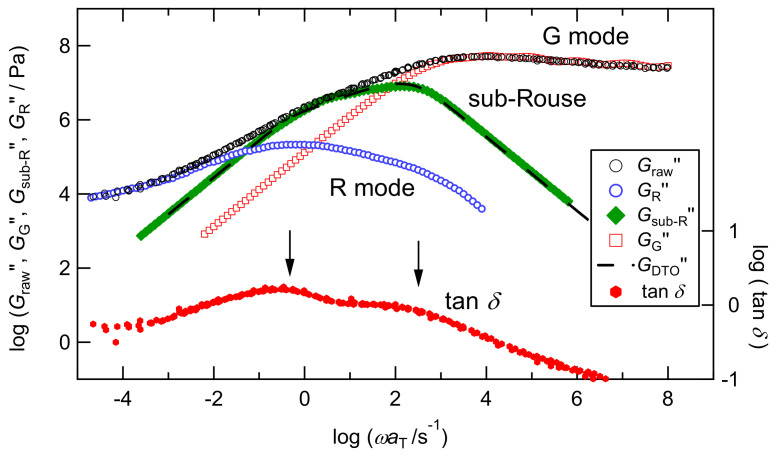
Reduced frequency *ω*a_T_ dependence of *G*″ (Graw″) and tan δ for molten PC_4_-TFSI at the reference temperature, 80 °C. Three-component *G*″ functions (*G*_R_″, *G*_sub-R_″, *G*_G_″) determined by modified-stress-optical-rule are overlayed on the raw data. The black dashed line represents the DTO model calculation used in the previous study [25].

Before discussing the dynamics of PC_4_-TFSI/LiTFSI mixtures in detail, we first review the hierarchical dynamics of the neat PC_4_-TFSI system reported previously. Figure 6 shows a typical viscoelastic spectrum, i.e., the angular frequency *ω* dependence of loss modulus *G*″(*ω*) (black circles) and tan δ(*ω*) (=*G*″(*ω*)/*G*′(*ω*); red circles), for the PC_4_-TFSI molten system reported previously [24]. Both curves shown here are superposed master curves at the reference temperature of 80 °C. We note that the time-temperature superposition principle held well, although it does not generally hold in the vicinity of the glass transition region of typical amorphous polymers [35]. In the previous study, we separated these spectra into three modes, rubbery (R), sub-Rouse, and glassy (G) modes, by analyzing the viscoelastic and strain-induced birefringence (rheo-optical) data based on the modified stress optical rule [24,25]. The determined three modes are displayed in this figure by blue circles for the R mode, green diamonds for the sub-Rouse mode, and red squares for the G mode. Among these three modes, the sub-Rouse mode was ascribed to the rotational motion of the TFSI anion in the early study [22]. However, because of its broad relaxation time distribution, the molecular mechanism has been reconsidered to be the torsional motion of the polymer chain enhanced by intramolecular cooperativity [24,25].

As for the sub-Rouse mode, we explained in the introduction that the intrachain bridge formation via the TFSI molecule (polycation-TFSI-polycation intramolecular bridge) [19], as schematically shown in Figure 2, enhances the intrachain torsional mode. It has been revealed that the sub-Rouse mode does not appear for smaller anions with low aspect ratio, such as BF_4_, and PF_6_ [24,25], probably because they are less likely to create the intramolecular bridge. We note that the damped torsional oscillator (DTO) model, having the same functional form as the Rouse model, successfully reproduced the sub-Rouse spectra as shown by the dashed line in Figure 6 [24,25].

The tan *δ* curve, also shown in this figure, exhibits two peaks. Since usual polymers have a single peak between the R and G modes and the low ω upturn in the tan δ curve, the two tan *δ* peaks in PC_4_-TFSI are well correlated with the existence of the sub-Rouse mode. It should be noted that the low *ω* peak locates in the transition region from the R to sub-Rouse mode and the high *ω* peak locates in the transition region from the sub-Rouse to G mode. This indicates that tracking the two tan δ peaks enables us to examine the sub-Rouse mode at least qualitatively. In the following, we investigate the effect of LiTFSI addition to PC_4_-TFSI on each dynamic mode using the G*(*ω*) and tan *δ*(*ω*) curves.

### 3.4. LiTFSI Concentration Dependence of the Viscoelastic Spectra

**Figure 7:**
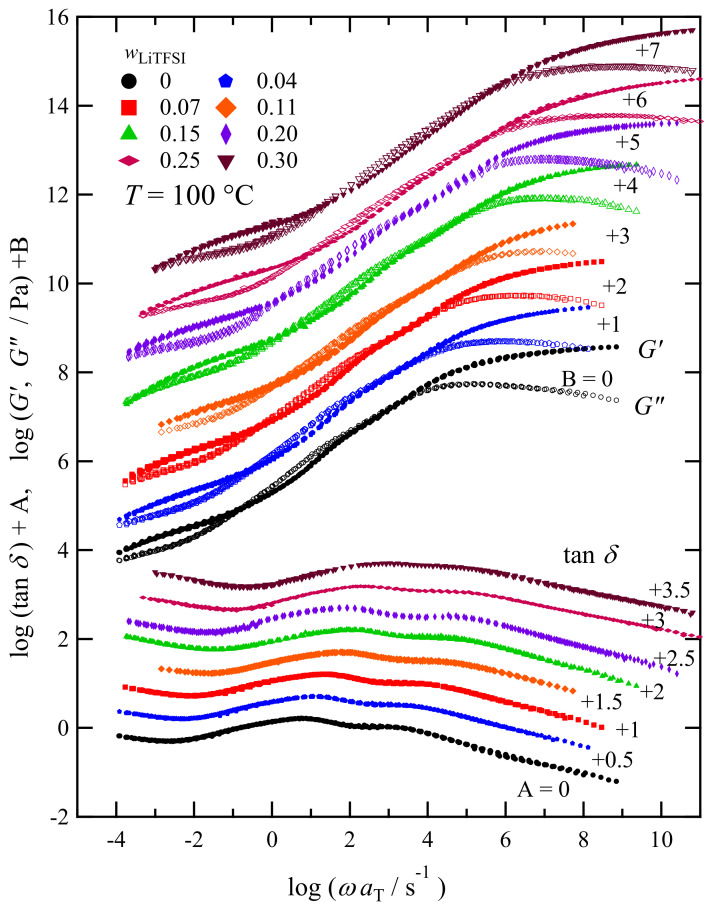
Angular frequency dependence of *G*′ (unfilled markers), *G*″ (filled markers), and tanδ for PC_4_-TFSI/LiTFSI mixtures with various LiTFSI concentrations at *T* = 100 °C. Each spectrum is vertically shifted with the factors B and A as indicated in the figure to avoid data overlapping.

Figure 7 shows the master curves of G* and tan *δ* for PC_4_-TFSI/LiTFSI mixtures with various LiTFSI weight fractions ($w_{\mathrm{LiTFSI}}$ ≤ 0.3) at a reference temperature 100 °C in the homogeneous state. The time-temperature superposition (tTS) principle worked well for these samples, which was also confirmed by van Gurp–Palmen plot as shown in Figure S1. To construct the master curves, first, we horizontally shifted the tanδcurve measured at each temperature onto the reference temperature data with the shift factor $a_{T}$. Second, the vertical shift with the shift factor *b*_T_ was also allowed for the superposition of the G* curves (in the form of G*b_T_), even though *b*_T_ is only the minor correction, as shown in Figure S2. While this superposition procedure worked well, we found the failure of tTS principle for the high $w_{\mathrm{LiTFSI}}$ samples (=0.35 and 0.40) due to the structural inhomogeneity, whose G*, tan δ, and van Gurp–Palmen plots are shown in Figure S3.

The viscoelastic spectra of homogeneously mixed PC_4_-TFSI/LiTFSI mixtures displayed in Figure 7 shift to the higher-frequency side with the increase in $w_{\mathrm{LiTFSI}}$, meaning that the addition of LiTFSI accelerates the entire relaxation processes of polymer chains and ions. This phenomenon is correlated with the decrease in *T*_g_. However, we see no significant difference in G* and tan *δ* between different concentrations in the spectral shapes. A detailed comparison of these spectral shapes is provided in Section 3.6.

**Figure 8:**
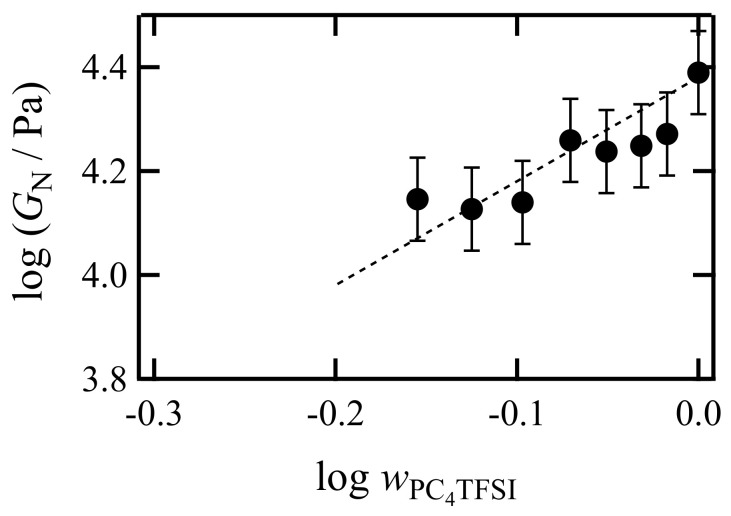
Dependence of plateau modulus *G*_N_ on the weight fraction of PC_4_-TFSI, $w_{{\mathrm{PC}}_{4}–\mathrm{TFSI}}$ for PC_4_-TFSI/LiTFSI mixtures. The dotted line represents a straight line with a slope of 2.

At the frequency of $\omega a_{T}$ = 10^−4^–10^−1^ s^−1^, *G′* exhibits the rubbery plateau region, indicating that the PC_4_-TFSI/LiTFSI mixtures in all the concentrations examined here are in a well-entangled state. The molecular weight of PC_4_-TFSI is 3.0 × 10^5,^ and the entanglement molecular weight *M*_e_ is reported to be 2.2 × 10^4^ [22]. Thus, considering *M*/*M*_e_ = 14, it is reasonable to observe a plateau region with a frequency range of about three orders of magnitude. From the *G′* spectra, we determined the plateau modulus *G*_N_ as the value of *G′* at the frequency where tanδshows a minimum in the rubbery plateau region at low *ω* [36]. Figure 8 displays the estimated values of *G*_N_ as a function of the weight fraction of PC_4_-TFSI, $w_{{\mathrm{PC}}_{4}\mathrm{TFSI}}$ (=$1-w_{\mathrm{LiTFSI}}$). In this double logarithmic plot, the dotted line indicates a linear relation with a slope of 2. For flexible entangled polymer solutions (polymer component dissolved in a low molecular weight solvent), the plateau modulus is known to be proportional to the 2–2.3 power of the polymer volume fraction *ϕ* [37,38]. Assuming that the volume fraction and the weight fraction are the same because the densities of PC_4_-TFSI and LiTFSI are similar, the *G*_N_ data being located on the dotted line implies that the addition of LiTFSI reduces the entanglement density of polymers due to the dilution effect in the similar manner as solvent molecules in ordinal polymer solutions. Thus our results demonstrate that LiTFSI can be regarded as merely a plasticizer for PC_4_-TFSI in the rubbery plateau region, and PC_4_-TFSI/LiTFSI mixtures behave like a flexible polymer solution system.

### 3.5. WLF Analysis of the Rheological Shift Factors

Figure 7 has demonstrated that the G* and tan δ curves shifted to the higher *ω* with increasing $w_{LiTFSI}$, reflecting the change in the glass transition temperature. The temperature dependence of the frequency shift factors $a_{T}$ used to construct the master curves involves essential information about the temperature dependence of the friction factor for the chain dynamics [39,40]. This section analyzes the shift factor data to examine the PIL chain dynamics under iso-frictional conditions.

**Figure 9:**
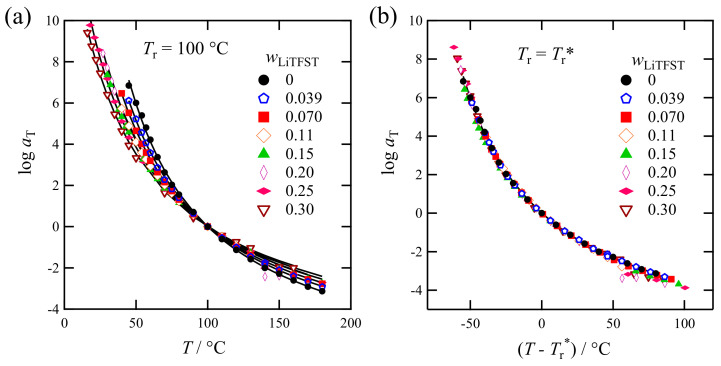
(**a**) Temperature dependence of the viscoelastic shift factors $a_{T}$ for the PC_4_-TFSI/LiTFSI mixtures at *T*_r_ = 100 °C. The solid curves represent the WLF functions. (**b**) *T* − *T*_r_* dependence of the shift factors $a_{T}$ at each reference temperature set to be *T*_r_* shown in Table 1.

Figure 9a plots the obtained value of $a_{T}$ for PC_4_-TFSI/LiTFSI mixtures at the reference temperature *T*_r_ = 100 °C as a function of the measurement temperature. The shift factor decreased with increasing temperatures, regardless of $w_{\mathrm{LiTFSI}}$. We fitted the $a_{T}$ curve of the neat PC_4_-TFSI ($w_{\mathrm{LiTFSI}}=0$) by the following Williams Landel Ferry (WLF) equation [39].

(3) $$\log a_{T}=\frac{C_{1}\left(T-T_{r}\right)}{C_{2}+T-T_{r}}$$

Here *C*_1_ and *C*_2_ are the parameters, and the values of *C*_1_ = 7.62 and *C*_2_ = 114 °C were obtained. According to the free volume theory, if the thermal expansion coefficient of the fractional free volume is the same, the *C*_1_ and *C*_2_ values become universal even for different *T*_g_ samples after a suitable choice of reference temperatures: *T*_r_ = *T*_g_ + constant [37]. Based on this assumption, we superposed the WLF curve fitted to the $w_{\mathrm{LiTFSI}}=0$ data onto the other data through the horizontal and vertical shifts. The detail of this method has been reported previously [41]. The fit results are shown by the solid lines in Figure 9a, demonstrating that a single WLF parameter set can approximately represent all the data.

The above operation enables us to determine an appropriate reference temperature (*T*_r_*) for each sample. Namely, by changing *T*_r_ (100 °C) to *T*_r_*, the $a_{T}$-curves can be superposed. The results are shown in Figure 9b, indicating that this $a_{T}$-superposition works well. Table 1 summarizes the obtained *T*_r_* values.

### 3.6. Chain Dynamics in Mixtures of PC_4_-TFSI/LiTFSI under Iso-Frictional Condition

**Figure 10:**
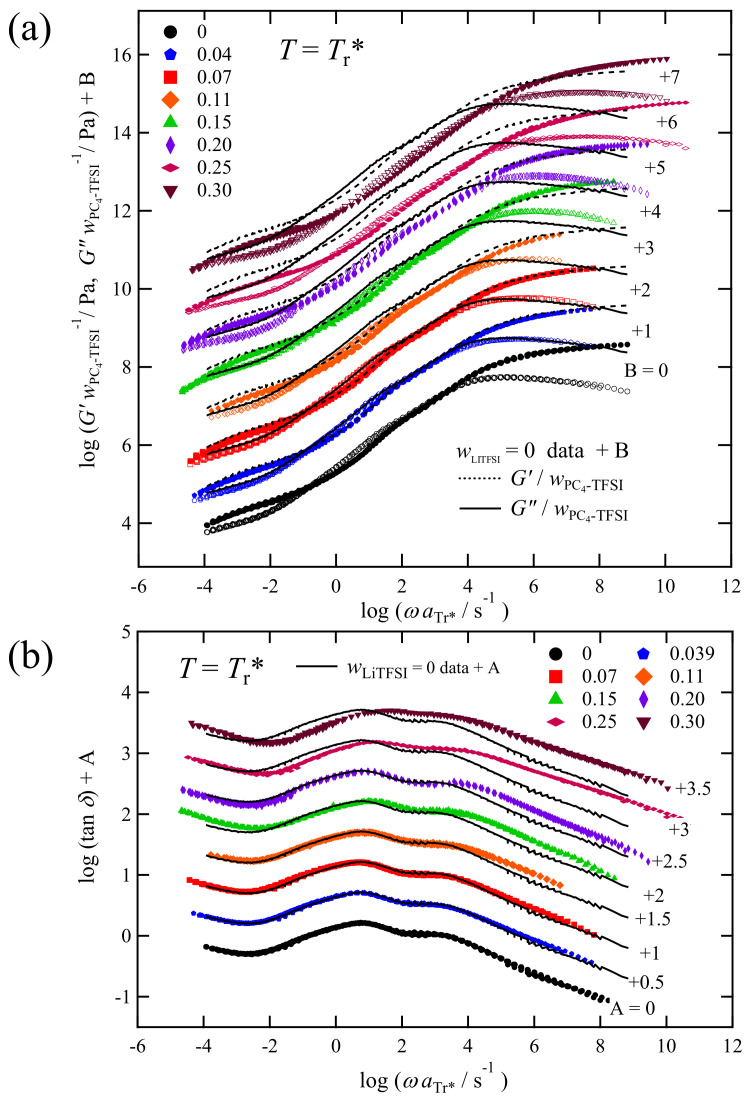
Angular frequency dependence of (**a**) $G^{*}/w_{{\mathrm{PC}}_{4}-\mathrm{TFSI}}$ and (**b**) tan *δ* for PC_4_-TFSI/LiTFSI mixtures with various LiTFSI concentrations at *T*_r_*. The data are vertically moved with the shift factors A and B indicated in the figure to avoid data overlapping. The solid and dotted lines represent the data of $w_{\mathrm{LiTFSI}}=0$ vertically shifted with the same factor A and B.

Figure 10a compares the *G*′ and *G*″ spectra normalized by the weight fraction of PC_4_-TFSI, $w_{{\mathrm{PC}}_{4}\mathrm{TFSI}}$ (=1 − $w_{\mathrm{LiTFSI}}$), at *T* = *T*_r_^*^, i.e., in the iso-frictional state (later, we will explain this is not an iso-frictional state). By assuming that the intensity of the Rouse dynamics inside the entanglement strands (involved in the R-mode) and that of more local chain dynamics is proportional to the weight concentration of the polymer component, these normalized curves enable us to compare the local chain dynamics of PC_4_-TFSI, including their intensities. Note that the normalization by $w_{\mathrm{LiTFSI}}$ is not valid in the plateau region as discussed in Figure 8 and for the tan *δ* (=*G*″$w_{\mathrm{LiTFSI}}^{-1}$/*G*′$w_{\mathrm{LiTFSI}}^{-1}$) spectra, shown in Figure 10b, this normalization is unnecessary. In these figures, the lines (solid and dotted lines) representing the data of $w_{\mathrm{LiTFSI}}=0$ are overlaid on each *G*′/$w_{{\mathrm{PC}}_{4}\mathrm{TFSI}}$, *G*″/$w_{{\mathrm{PC}}_{4}\mathrm{TFSI}}$, and tanδ curves as reference spectra for comparison. At low $w_{\mathrm{LiTFSI}}$ (≤0.07), the shapes of G* and tan *δ* are almost the same as the reference data of $w_{\mathrm{LiTFSI}}=0$. However, as $w_{\mathrm{LiTFSI}}$ increases, the deviation, especially in the high-frequency region, becomes more prominent. Eventually, at the highest $w_{\mathrm{LiTFSI}}\left(=0.3\right)$ examined, the values of *G*′/$w_{\mathrm{LiTFSI}}$ and *G*″/$w_{{\mathrm{PC}}_{4}\mathrm{TFSI}}$ always become smaller than those of the reference curve over the wide frequency range, including the Rouse dynamics region just above the rubbery plateau section observed in a frequency range of −1 ≤ log(*ω*$a_{T_{r}^{*}}$/*s*^−1^) ≤ 2.

In general, the part of the viscoelastic spectra responsible for the Rouse dynamics should be superposed in this type of plot if compared at the reference temperature *T*_r_* obtained from the WLF analysis. The deviation of the G^*^ spectra in the Rouse region implies that the reference temperatures (*T*_r_^*^) do not provide an iso-frictional condition for some reason. In Section 3.5, we assumed that *C*_1_ and *C_2_* values become universal if *T*_r_* = *T*_g_ + constant is satisfied. This assumption means that the temperature dependence of the free-volume fraction (the thermal expansion factor for free volume) is the same by taking *T*_g_ as a standard point. We speculate that this assumption may not be valid for the PC_4_-TFSI/LiTFSI mixtures. For example, in a multi-component system of cations and anions, various Coulomb interactions could cause a spatial distribution in the free volume fraction. They can change the thermal expansion coefficient for free volume. In such a case, the universality of the *C*_1_ and *C*_2_ values may not hold.

**Figure 11:**
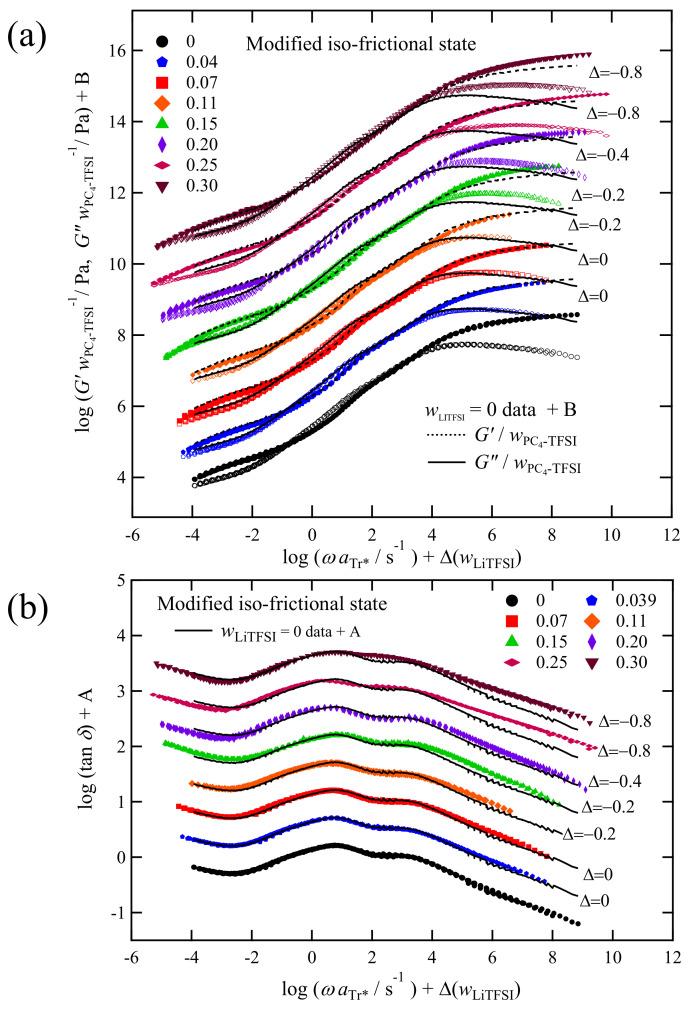
Comparison of (**a**) $G^{*}/w_{{\mathrm{PC}}_{4}-\mathrm{TFSI}}$ and (**b**) tan *δ* in the modified iso-frictional state for PC_4_-TFSI/LiTFSI mixtures with various LiTFSI concentrations. The A and B values are the same as those shown in Figure 10. Horizontal shift factors (Δ) depending on $w_{\mathrm{LiTFSI}}$ are shown in the figure.

**Figure A1:**
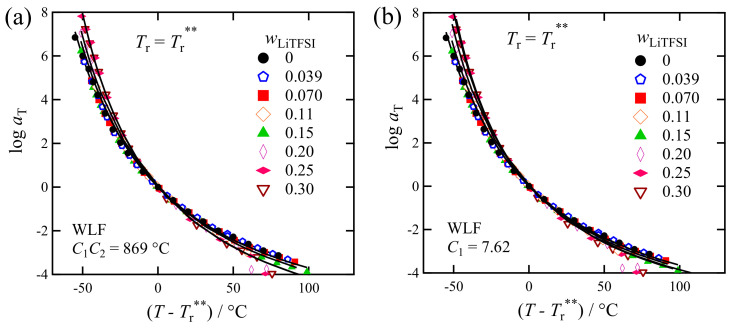
*T* − *T*_r_^**^ dependence of the shift factors with the reference temperature *T*_r_^**^ shown in Table 1. The solid curves in figure (**a**) represent the WLF functions given by Equation A1 with the parameter sets shown in Table A1. The solid curves in figure (**b**) also represent the WLF functions with different parameter sets, i.e., *C*_1_ was fixed to be 7.62, and *C*_2_ was varied (see Table A1).

To correct the difference in the friction factors in the present analysis, we apply a rather simple method as follows: shifting the entire viscoelastic spectrum by a factor of Δ along the direction of the horizontal axis such that the data at log(ω$a_{T_{r}^{*}}$/s^−1^) = −1–2 (the Rouse region) overlap each other. Figure 11 shows these shifting results along with the values of the factor Δ. This operation is equivalent to taking new reference temperatures (*T*_r_**) to compare the viscoelastic spectra in the iso-frictional state. The values of *T*_r_** calculated from the Δ values are provided in Table 1. The difference between *T*_r_* and *T*_r_** reflects that the *C_1_* and *C_2_* in the WLF function change with $w_{\mathrm{LiTFSI}}$. In other words, the fragility [42] of the mixture is dependent on $w_{\mathrm{LiTFSI}}$. Temperature dependence of the shift factors with the new reference temperature (*T*_r_**) along with the (*C_1_*, *C_2_*) parameter sets are shown in Figure A1, and Table A1, indicating that the functional forms of *a*_T_ with *T*_r_ = *T*_r_** are weakly dependent on $w_{\mathrm{LiTFSI}}$. In Appendix A, we discuss the physical meaning of the slight difference in the temperature dependence of the shift factors—we concluded that the thermal expansion factor for free volume could change with $w_{\mathrm{LiTFSI}}$.

By using the new WLF equation (Equation (A1)) with *T*_r_ = *T*_r_** (cf. *C*_2_ with fixed *C*_1_ in Table A1), we can estimate the glass transition temperatures. Since $a_{T}$ is expressed by the ratio of the relevant relaxation times (τ), $a_{T}=\tau \left(T\right)/\tau \left(T_{r}^{**}\right)$, the *a*_T_ value at $T=T_{g}$ should be the same for all the samples. We obtained $\log a_{T}=4.793$ at $T=T_{g}$ for neat PC_4_-TFSI by using its *T*_g_ value (=56 °C). For the other PC_4_-TFSI/LiTFSI mixtures, *T*_g_ values can be calculated to give $\log a_{T}=4.793$. Thus determined *T*_g_s, denoted as *T*_g_(WLF), have been already shown in Figure 5, indicating that *T*_g_(DSC) and *T*_g_(WLF) are similar. Considering that *T*_g_(WLF) mainly reflects the dynamics of PC_4_-TFSI from its determination method, the proximity of *T*_g_(DSC) and *T*_g_(WLF) suggests that the heat capacity of the polymer component dominantly determine the thermal glass transition for PC_4_-TFSI/LiTFSI mixtures.

Figure 11 shows that all the spectra of both G* and tan *δ* are in close agreement with the reference curve in the frequency range below 10^4^ s^−1^ where the Rouse dynamics is dominant. As explained in Section 3.3, the transition of the relaxation modes from the sub-Rouse to G modes appears at the frequency region around the high-ω peak in the spectra of tan *δ*. In Figure 11b, such a peak locates at around 10^4^ s^−1^, below which both G* in Figure 11a and tan *δ* in Figure 11b exhibit the same dependence on frequency, indicating that the R-mode and sub-Rouse mode are independent of the weight fraction of LiTFSI. Namely, the relaxation mechanism of PC_4_-TFSI chain is not strongly modified by the addition of LiTFSI. As explained earlier, the sub-Rouse mode is assumed to emerge if the low interchain and high intrachain cooperativity of chain segments are satisfied. Our intuition was that the addition of LiTFSI might decrease the interchain cooperativity due to the dilution effect, and thus enhancing the sub-Rouse mode. However, Figure 11, i.e., almost no concentration dependence in the sub-Rouse region, indicates that the dilution effect less contributes to the appearance of the sub-Rouse mode in this system. One of the possible reasons is that since PILs originally contain counterion in the same molar ratio to monomers, some of the dissociated counterions substantially self-dilute the system without adding LiTFSI. As for intramolecular cooperativity, the formation of the intramolecular bridge-like coordination, as depicted in Figure 2, is hypothesized to be necessary to trigger the sub-Rouse motions. It is reasonable to consider that such local coordination structures do not change much even when LiTFSI is doped. The MD simulation [19] indicated that there could be various aggregation structures, including the co-coordination of polycation–TFSI(anion)–Polycation, polycation–TFSI(anion)–Li(cation), etc., and their fractions vary with the salt concentration. Such variation of the coordination structure could induce the change in the sub-Rouse mode. However, we deduce that our results do not contradict these MD results because our data merely show that the local association (coordination) structure, effective for the sub-Rouse mode, does not change much.

Finally, we focus on the high-frequency (>10^4^ s^−1^) response of the G-mode region in Figure 11. Under the modified iso-frictional state using *T*_r_**, we can see that the height of G*/$w_{{\mathrm{PC}}_{4}\mathrm{TFSI}}$ increases with increasing $w_{\mathrm{LiTFSI}}$, and correspondingly the tan δ curves at the high-frequency side also become broader. In particular, such a trend is more pronounced for the two highest $w_{\mathrm{LiTFSI}}$ systems ($w_{\mathrm{LiTFSI}}$ = 0.25 and 0.30). According to the MD simulation [19], the increase of the LiTFSI concentration leads to the formation of some Li–TFSI coordinated species free from the polymer backbone, such as Li(TFSI)_3_, in addition to free TFSI anions. These species may work as plasticizers of the polymer component, i.e., behave as dynamically fast components, resulting in the dynamically heterogeneous behaviors, such as the broadening of the G* and tan *δ* spectral shapes and the appearance of prominent fast component dynamics in the high *ω* region [43,44,45,46]. Specifically, by looking at the high-frequency region of *ω* > 10^6^ s^−1^ for the $w_{\mathrm{LiTFSI}}$ = 0.25 and 0.30 data, the slopes of tan *δ* (and *G*″) are weaker than those at smaller $w_{\mathrm{LiTFSI}}$, suggesting the existence of the fast relaxation component in the viscoelastic spectra.

The conclusion in this section is that the addition of LiTFSI hardly affects the sub-Rouse mode but causes an increase in the relative intensity of the G-mode and broadening of the spectrum. The latter result suggests that fast dynamics of low molecular weight components, such as free TFSI and coordinated ones with Li^+^, occur in the glassy region.

## 4. Conclusions

We conducted linear viscoelastic measurements on the PC_4_-TFSI/LiTFSI mixtures to examine the effect of the salt additives on hierarchical chain dynamics. The findings of this study are as follows. (1) LiTFSI was homogeneously dissolved in PC_4_-TFSI in the range of the LiTFSI weight fraction $w_{\mathrm{LiTFSI}}$ below 0.3. (2) The glass transition temperature decreased with increasing the LiTFSI concentration, and the Fox equation could reproduce its concentration dependence. (3) In a homogeneous PC_4_-TFSI/LiTFSI mixture ($w_{\mathrm{LiTFSI}}$ ≤ 0.3), the time-temperature principle worked well. (4) The height of the rubbery plateau modulus was regarded to be proportional to the square of the PC_4_-TFSI concentration. This result indicated that LiTFSI acts as a solvent molecule similarly in typical polymer solution systems. (5) Comparison of the viscoelastic spectra at the reference temperature *T*_r_* determined by the WLF analysis did not give the iso-frictional condition for PC_4_-TFSI, suggesting a failure of our assumption that the free volume expansion factor is the same, independent of *w*_LiTFSI_. (6) We proposed a simple way to compare the viscoelastic data in the iso-frictional state by superposing the $G^{*}/w_{\mathrm{LiTFSI}}$ data in the frequency domain and obtained new reference temperature *T*_r_**. (7) The temperature dependence of the viscoelastic shift factor becomes weaker with increasing $w_{\mathrm{LiTFSI}}$ if compared at the newly determined reference temperature *T*_r_** indicating that the *C*_1_ and *C*_2_ values are dependent on $w_{\mathrm{LiTFSI}}$. (8) The stress relaxation mechanism of PC_4_-TFSI chains, including the sub-Rouse mode, was the same independent of $w_{\mathrm{LiTFSI}}$ under the iso-frictional condition. (9) At $w_{\mathrm{LiTFSI}}$ = 0.25 and 0.30, the frequency dependence of *G*″ and tan δ on the high-frequency side become weaker, resulting in broader spectra than other concentrations. This result suggested that the response of low molecular weight components, e.g., free TFSI anions and some kinds of Li–TFSI coordinated species decoupled from polymer chain motion, could be observed at high $w_{\mathrm{LiTFSI}}$.

This study demonstrated the usefulness of the rheological analysis to examine the dynamics of the polymeric component and the response of the low molecular weight components. This method will be one of the practical tools to analyze the dynamic properties of polymer electrolytes, e.g., ion mobilities, mechanical toughness, and their temperature dependencies, etc. We will report the results on the ionic conductivity of this system and the relation with the rheological data in the near future.

## Figures and Tables

**Table 1:** Reference temperatures, *T*_r_* and *T*_r_**, respectively determined from the WLF analysis and superposition of G* spectra for PC_4_-TFSI/LiTFSI mixtures with various $w_{\mathrm{LiTFSI}}$.

$w_{\mathrm{LiTFSI}}$	*T*_r_*/°C	*T*_r_**/°C
0	100	100
0.039	93.9	93.9
0.07	89.2	89.2
0.11	83.8	80.9
0.15	84.1	81.1
0.20	83.7	78.0
0.25	79.5	68.7
0.30	75.1	64.3

## Data Availability

Not applicable.

## Supplementary Materials

The following are available online at https://www.mdpi.com/article/10.3390/polym13111772/s1. Figure S1: Van Gurp–Palmen plot for PC_4_-TFSI/LiTFSI mixtures with *w*_LiTFSI_ ≤ 0.3 at the reference temperature, 100 °C; Figure S2: Temperature dependence of the vertical shift factor *b*_T_ for PC_4_-TFSI/LiTFSI mixtures with *w*_LiTFSI_ ≤ 0.3 at the reference temperature, 100 °C; Figure S3: (a) Reduced frequency *ωa*_T_ dependence of *G*′, *G″* and tan *δ* curves at the reference temperature, 100 °C, and (b) the corresponding van Gurp–Palmen-plot for PC_4_-TFSI/LiTFSI mixtures with *w*_LiTFSI_ = 0.35 and 0.40.

## Appendix A

Figure A1a shows the temperature dependence of the viscoelastic shift factors (*a*_T_) with the reference temperatures *T*_r_^**^ determined from the superposition of the $G^{*}/w_{{\mathrm{PC}}_{4}\mathrm{TFSI}}$ spectra as explained in Figure 11. We can see that *T* − *T*_r_^**^ dependence of *a*_T_ of each *w*_LiTFSI_ data does not fall on a single universal curve. These *w*_LiTFSI_ dependent *a*_T_ vs. *T* − *T*_r_^**^ curves indicates that the different parameter set (*C*_1_ and *C*_2_) in the WLF equation is necessary for each *w*_LiTFSI_ data. Actually, the solid curves in this figure are a single WLF curve with *C*_1_ = 7.62 (≡*C*_1_^0^) and *C*_2_ = 114°C (≡*C*_1_^0^) (corresponding to the neat PC_4_-TFSI data) shifted vertically and horizontally and superposed on each data point. This procedure can give the new *C*_1_ and *C*_2_ values cast into the following equation.

(A1) $$\log a_{T}=\frac{C_{1}\left(T-T_{r}^{**}\right)}{C_{2}+T-T_{r}^{**}}$$

The following equations give the *C*_1_ and *C*_2_ values in Equation (A1).

(A2) $$C_{1}=\frac{C_{1}^{0}C_{2}^{0}}{C_{2}^{0}+T_{r}^{**}-T_{r}^{*}}\;,\;\;C_{2}=C_{2}^{0}+T_{r}^{**}-T_{r}^{*}$$

Table A1 summarizes the *C*_1_ and *C*_2_ values calculated with Equation (A2).

According to the free volume theory, *C*_1_ and *C*_2_ are related to the free volume parameters:

(A3) $$C_{1}=0.4342\frac{B}{f_{0}},\;\;C_{2}=\frac{f_{0}}{\alpha_{f}}$$

Here, *f*_0_ is the fractional free volume at the reference temperature, *α*_f_ is the thermal expansion factor for free volume, and *B* is a constant of order unity [37,38]. The product of $C_{1}C_{2}\left(=0.4342B\alpha_{f}^{-1}\right)$ is proportional to $\alpha_{f}^{-1}$. Since Equation (A2) gives $C_{1}C_{2}=C_{1}^{0}C_{2}^{0}(=869\;{}^{\circ}C$) which is confirmable from the values listed in Table A1, meaning that the thermal expansion factor is independent of *w*_LiTFSI_, the fractional free volume *f*_0_ at *T* = *T*_r_^**^ is a function of *w*_LiTFSI_. This leads to a physical contradiction because the iso-frictional and iso-free volume states are different even though the rheological measurement detects the same PC_4_-TFSI dynamics. Therefore, we fix the *C*_1_ value (fixing *f*_0_) and change the *C*_2_ value to refit the *a*_T_ data. The results are shown in Figure A1b by the solid curves. The fitting qualities are not so different from those shown in Figure A1a. The variation of *C*_2_ corresponds to the change of *α*_f,_ whose values can be calculated by assuming *B* = 1 in Equation (A3). Table A1 also shows the new *C*_2_ and calculated *α*_f_ values. Since the Coulomb interaction among the three ionic species, Li^+^, TFSI^−^, and PC_4_^+^, varies with *w*_LiTFSI_, we believe that *α*_f_ being dependent on the concentration is a characteristic feature of this system. We have reported that such a change in *α*_f_ does not occur in miscible polymer blends with hydrogen bonding interaction weaker than the Coulomb interaction [47,48], suggesting that the intermolecular interaction strength could be crucial.

**Table A1:** *C*_1_ and *C*_2_ values calculated from Equation (A2), *C*_2_ values determined by fitting with fixed *C*_1_ (=7.62), and thermal expansion factor *α*_f_ for free volume calculated from Equation (A3).

$w_{\mathrm{LiTFSI}}$	*C*_1_ (Equation (A2))	*C*_2/_°C (Equation (A2))	*C*_2/_°C with *C*_1_ = 7.62	10^4^ *α*_f_/°C^−1^
0	7.62	114	114	5.00
0.039	7.62	114	114	5.00
0.07	7.62	114	114	5.00
0.11	7.82	111	109	5.23
0.15	7.82	111	109	5.22
0.20	8.02	108	103	5.53
0.25	8.42	103	97.0	5.88
0.30	8.42	103	96.0	5.94
