# Supplementary material for: Viscoelastic Relaxation of Polymerized Ionic Liquid and Lithium Salt Mixtures: Effect of Salt Concentration
Source: Polymers (Basel). 2021 May 28;13(11):1772. doi: 10.3390/polym13111772 (PMC8199314; doi:10.3390/polym13111772)
Supplement: Supplementary file 1 [file polymers-13-01772-s001.zip › polymers-1225027-supplementary.pdf]

Supplementary Information for

Viscoelastic Relaxation of Polymerized Ionic Liquid and Lithium Salt Mixtures: Effect of Salt Concentration

Arisa Yokokoji[1], Wakana Kitayama[1], Kamonthira Wichai[1,2], Osamu Urakawa[1*], Atsushi Matsumoto[3], Visit Vao-soongnern[2], Tadashi Inoue[1*]

[1]Department of Macromolecular Science, Graduate School of Science, Osaka University, 1-3 Machikaneyama-cho, Toyonaka, Osaka 560-0043, Japan

[2]School of Chemistry, Institute of Science, Suranaree Universitty of Technology, NakonRatchasima 30000, Thailand

[3] Department of Applied Chemistry and Biotechnology, University of Fukui, 3-9-1 Bunkyo, Fukui-shi, Fukui 910-8507, Japan

*Correspondence: Email: urakawa@chem.sci.osaka-u.ac.jp;

Email: tadashi@chem.sci.osaka-u.ac.jp;

1. Van Gurp-Palmen-Plot

For the verification of the time-temperature superposition principle, we plotted the phase angle δ of the measured rheological data against the corresponding absolute value of the complex shear modulus G^*, so-called van Gurp-Palmen-plot, for all the w_{LiTFSI} mixtures. The data measured at various temperatures converge in a single curve indicating the validity of the time-temperature superposition principle.

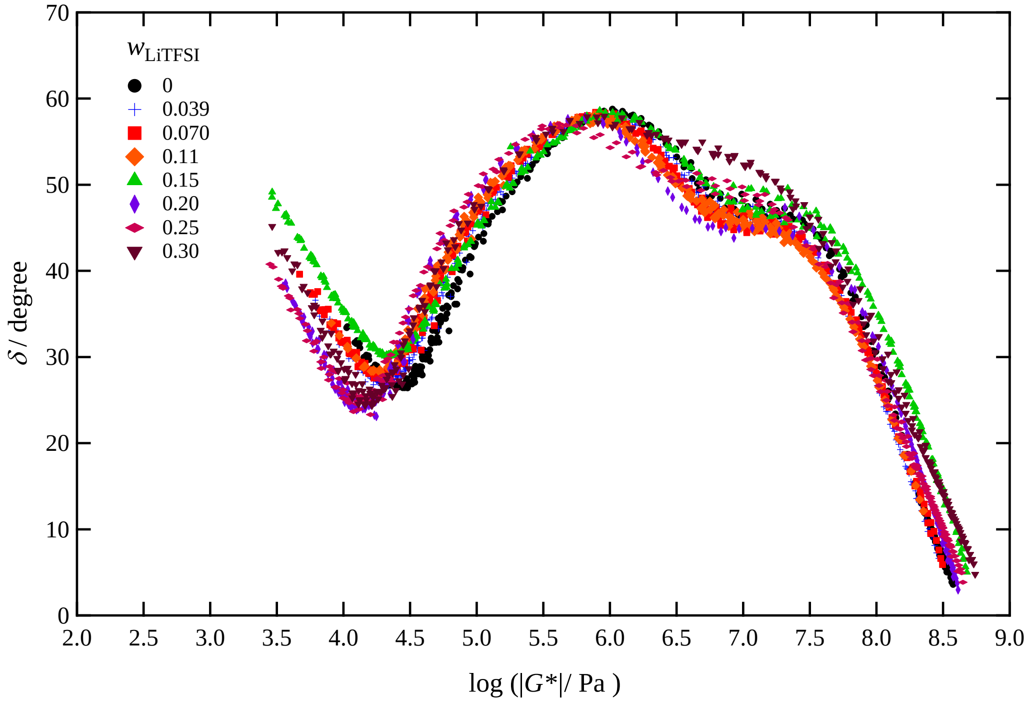

Figure S1 Van Gurp-Palmen plot for PC_4-TFSI / LiTFSI mixtures with w_{LiTFSI} ≤0.3.

2. Vertical shift factors

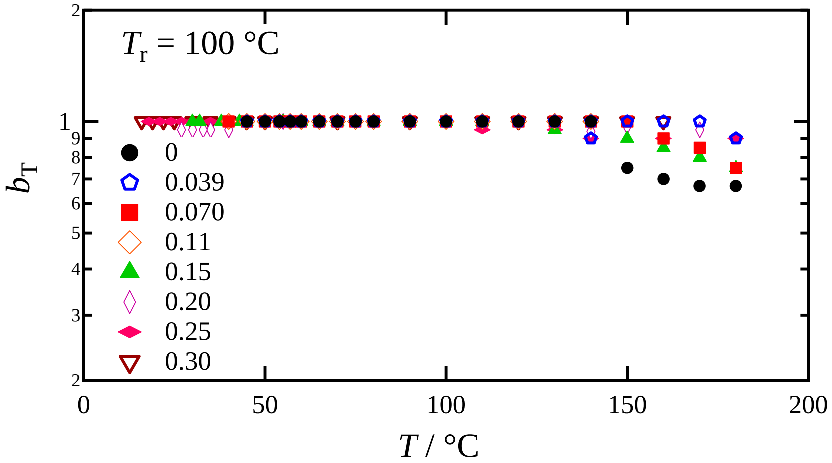

Figure S2 Temperature dependence of the vertical shift factor b_T for PC_4-TFSI / LiTFSI mixtures with w_{LiTFSI} ≤0.3 at the reference temperature,100°C.

3. Failure of Time-Temperature Superposition Principle

For the inhomogeneous mixtures (w_{LiTFSI} = 0.35 and 0.40), the time-temperature superposition principle (tTS) fails. Figure S2(a) shows the master curves of G^* and tan δ constructed using only the superposable part. The tTS holds for the high-temperature and low-frequency data but fails for the low-temperature and high-frequency data (especially in the tan δ data). Figure S2(b) displays van Gurp-Palmen-plot, which clearly shows the failuar of tTS. These results suggest that the temperature change induced the structural change, e.g. change of the degree of crystallinity.

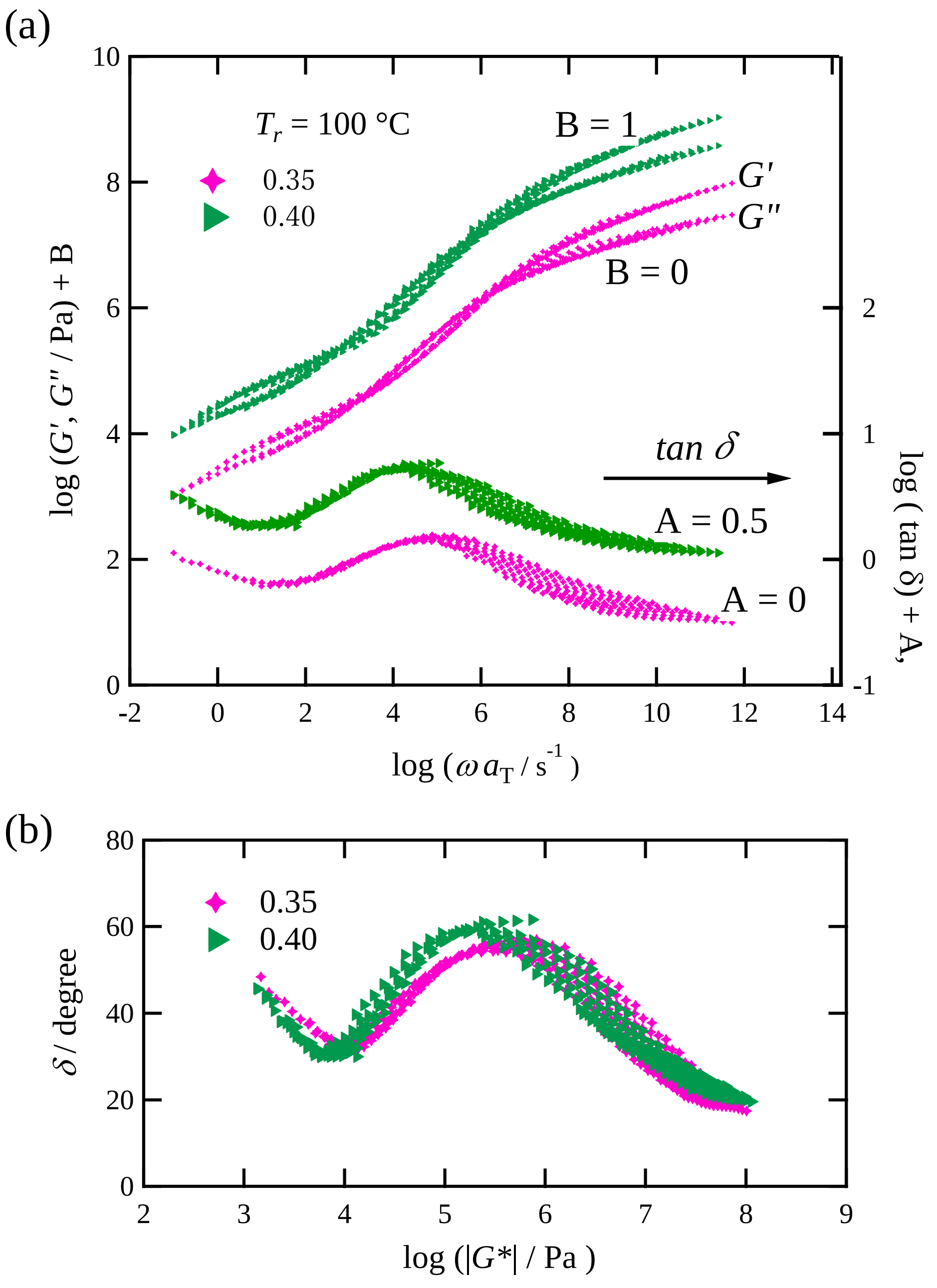

Figure S3 (a) Reduced frequency ωa_T dependence of G', G'' and tan δ curves at the reference temperature,100°C,.and (b) the corresponding van Gurp-Palmen-plot for PC_4-TFSI / LiTFSI mixtures with w_{LiTFSI} = 0.35 and 0.40
